# Evaluating the Association Between Iron Deficiency Anemia and Febrile Convulsion Among Children Aged 6-60 Months Admitted to a Tertiary Care Hospital in Eastern India: A Case-Control Study

**DOI:** 10.7759/cureus.58761

**Published:** 2024-04-22

**Authors:** Sandip K Mandal, Saumyen DE, Rashmita Das, Nikahat J Awati, Nilanjana Dey, Abhijit Biswas

**Affiliations:** 1 Paediatrics, College of Medicine & Sagore Dutta Hospital, Kolkata, IND

**Keywords:** iron deficiency anemia (ida), febrile convulsion, children, rbc indices, parental anxiety

## Abstract

Background and objective

Anemia, particularly iron deficiency anemia (IDA), presents a significant global health challenge, particularly among children under the age of five years in developing nations. Concurrently, febrile convulsions (FC) affect up to 5% of neurologically healthy children aged 6-60 months, causing considerable distress among parents. There is a suggested correlation between fever and iron deficiency, which may exacerbate neurological risks, potentially lowering seizure thresholds and increasing the risk of FC. However, studies investigating the relationship between IDA and FC have shown conflicting results. In light of this, this study aimed to explore this relationship among children aged 6-60 months in Eastern India, an area where this association has yet to be thoroughly investigated.

Materials and methods

The case-control study included children aged 6-60 months. The cases consisted of children presenting with FC, while controls comprised children in the same age group presenting with febrile illness but without seizures. Informed consent was obtained, a detailed history was taken, and clinical examinations were conducted for both groups. Blood investigations were performed to diagnose IDA according to WHO criteria: hemoglobin <11 gm/dl with the classical triad of low mean corpuscular volume (MCV), low mean corpuscular hemoglobin (MCH), and low mean corpuscular hemoglobin concentration (MCHC) for age. Data analysis was performed using the R-based software Jamovi 2.4.8. with appropriate statistical tests.

Results

We included 81 cases and 80 controls. The study found a statistically significant association between IDA and FC with an odds ratio (OR) of 2.25 [95% confidence interval (CI): 1.03-4.91; p=0.039]. Additionally, the study revealed that hemoglobin levels, MCH, MCV, and MCHC were lower among cases compared to controls, while the red cell distribution width (RDW) was higher. Both these findings regarding RBC indices were statistically significant (p<0.05).

Conclusions

Our findings indicate a statistically significant association between IDA and FC among children under five years of age. Implementing measures to prevent IDA and strengthening existing strategies may help alleviate the burden of FC in this vulnerable population.

## Introduction

Anemia poses a pressing healthcare concern on a global scale, with children under the age of five in developing countries bearing the brunt of its impact [1]. Among the various forms of nutritional deficiency anemia, iron deficiency anemia (IDA) accounts for a significant portion of this global burden [2,3]. Meanwhile, up to 5% of neurologically healthy children aged 6-60 months experience at least one episode of febrile convulsion (FC), causing significant parental distress [4]. Fever exacerbates the adverse effects of an iron-deficient state on the central nervous system, potentially lowering the seizure threshold in children with IDA and increasing the risk of convulsions [5].

Although the relationship between IDA and FC has been investigated in various settings, the conclusions drawn from different studies are not consistently aligned [6-8], with some showing conflicting results. Hence, we conducted this study to determine the association between IDA and FC among children aged 6-60 months in the eastern region of India, where this relationship remains relatively unexplored. Additionally, we aim to provide clarity on the conflicting findings in the existing literature regarding this topic.

## Materials and methods

Study design, setting, and participants

This case-control study was conducted in the Pediatrics Department of the College of Medicine & Sagore Dutta Hospital from January 2023 to December 2023, after obtaining approval from the Institutional Ethics Committee (IEC) of the College of Medicine & Sagore Dutta Hospital, as indicated by the clearance certificate bearing memo number CMSDH/IEC/327/10-2022, dated October 22, 2022. The study was registered under IEC (Regn No: ECR/1210/Inst/WB/2019/RR-22).

Assuming an alpha error of 5% and a study power of 80%, the sample size was calculated using the Epi-info program. According to the National Family Health Survey 2019-21 (NFHS-5), the prevalence of anemia among children aged 6-60 months in West Bengal is 69% [1], with approximately 50% attributed to iron deficiency based on previous WHO reports and studies [2,3]. Thus, the prevalence of exposure (iron deficiency) in the control (non-convulsive control) group was estimated to be 35%. The odds ratio (OR), obtained from a study published by Chaudhury et al. [9] in 2021 provided a value of 76 children in each group. Accounting for a non-response rate of 5%, the calculated final sample size in each arm was 80. Consequently, the total sample size for the study was determined to be 160.

During the study period, children aged 6-60 months admitted with FC were categorized as cases. The diagnostic criteria for FC [4] were as follows: seizures accompanied by fever without evidence of central nervous system infection, metabolic or electrolyte imbalances, a history of afebrile seizures, or any acute neurological injury/head trauma in children aged 6-60 months. The control group comprised children aged 6-60 months with short febrile illnesses (<5 days duration) without any history of convulsions in the current episode or the past. We ensured a 1:1 ratio for cases and controls, and consecutive cases and controls were enrolled and included in the study after getting informed consent from their parents/guardians. Children presenting with afebrile seizures, exhibiting signs of central nervous system infections, having chronic neurologic disorders, those previously diagnosed with other hematologic problems (such as thalassemia, coagulation disorders, or hematological malignancies), or currently taking anti-epileptic medications, as well as critically ill children, were excluded from the study.

Data collection

Following inclusion, a detailed history was obtained, and a physical examination was performed for every child in both groups as per the pre-designed proforma. Relevant data were also collected from hospital records. Laboratory investigations for diagnosing IDA included hemoglobin (Hb), red blood cell (RBC) indices such as mean corpuscular hemoglobin (MCH), mean corpuscular volume (MCV), mean corpuscular hemoglobin concentration (MCHC), and red cell distribution width (RDW). These tests were performed using an automated hematology analyzer (Sysmex Kx -21). However, due to logistical issues, serum iron studies, i.e., serum iron, total iron binding capacity (TIBC), and transferrin saturations, were not performed. IDA was diagnosed according to WHO criteria, which includes a Hb level of less than 11 gm/dl, along with the classical triad [10] of low MCV (<75 fl), low MCH (<27 pg), and low MCHC (<32%) for children aged <5-years.

Statistical analysis

Statistical analysis was conducted using the R-based software Jamovi 2.4.8. The Shapiro-Wilk test was employed to assess whether the samples drawn from the population followed a normal distribution for different study variables. For normally distributed variables, an independent t-test was applied. For variables that were not normally distributed, the Mann-Whitney U (MHU) test was used. To compare the proportions of anemia and IDA between the two groups, the Chi-square test was employed. A p-value <0.05 was considered statistically significant.

## Results

In this study, a total of 97 potential cases and 100 potential controls were initially reviewed; 81 cases and 80 controls were ultimately included for the final analysis (case-control ratio of 1.01:1). Reasons for exclusion of 13 potential cases were as follows: met exclusion criteria (10), missing data (3), second-time presentation during study period resulting in duplication of patients (3). Potential 20 controls were excluded for the following reasons: parents refused to give consent (12), and missing data (8). The mean age of the cases was 26 ± 14.4 months, while 25.8 ± 13.5 months for controls. Out of 81 cases, 49 (60.5%) were male and 32 (39.5%) were female, whereas among controls, 49 (61%) were male and 31 (39%) were female. Additionally, 76 (94-95%) children were born at term in both groups. The history of FC and epilepsy among first-degree relatives was higher in cases compared to controls (Table 1).

**Table 1:** The association of various categorical variables with febrile convulsion among the participants A p-value <0.05 is considered statistically significant CI: confidence interval

Variables	Cases (n=81), n (%)	Controls (n=80), n (%)	Odds ratio (95% CI)	P-value
Male	49 (60.5%)	49 (61%)	0.97 (0.51-1.82)	0.922
Female	32 (39.5%)	31 (39%)		
Term	76 (94%)	76 (95%)	0.8 (0.2-3.09)	0.746
Preterm	5 (6%)	4(5%)		
History of febrile convulsion among first-degree relatives	21 (25.9%)	12 (15%)	1.98 (0.9-4.37)	0.08
History of epilepsy among first-degree relatives	17 (20.9%)	10 (12.5%)	1.86 (0.79-4.36)	0.15

In the case group, 39 (48.1%) children were anemic, while 30 (37.5%) children were anemic in the control group (OR: 1.55, 95% CI: 0.83-2.9); however, this difference was not found to be statistically significant (p=0.17) (Table 2).

**Table 2:** Proportion of anemia among cases and controls A p-value <0.05 is considered statistically significant Hb: hemoglobin; CI: confidence interval

Anemic (Hb <11 gm/dl)	Cases, n (%)	Controls, n (%)	Odds ratio (95% CI)	P-value
Yes	39 (48.1%)	30 (37.5%)	1.54 (0.83-2.9)	0.17
No	42 (51.9%)	50 (62.5%)		

However, concerning IDA, our study revealed that 23 children (28.4%) among cases had IDA, whereas, among controls, the number of children with IDA was 12 (15%), showing that the proportion of IDA was higher among cases than in the control group [OR: 2.25, 95% confidence interval (CI): 1.03-4.91; p=0.039]; this difference was statistically significant (Table 3).

**Table 3:** Proportion of IDA among cases and controls A p-value <0.05 is considered statistically significant CI: confidence interval; IDA: iron deficiency anemia

IDA	Cases, n (%)	Controls, n (%)	Odds ratio (95% CI)	P-value
Present	23 (28.3%)	12 (15%)	2.25 (1.03–4.91)	0.039
Absent	58 (71.7%)	68 (85%)		

We also found that hemoglobin levels, MCH, MCV, and MCHC were lower among cases than in controls. Conversely, the RDW was higher among cases than controls. Both these findings were statistically significant (p<0.05). Additionally, our study revealed that the RBC count and hematocrit levels were lower among cases than controls; however, the difference was not found to be statistically significant.

**Table 4:** The association of various continuous variables with febrile convulsion among the participants *The independent t-test was applied to normally distributed variables, while the Mann-Whitney U test was used for other variables where data did not suggest normality in the population values A p-value <0.05 is considered statistically significant SD: standard deviation; Hb: hemoglobin; HCT: hematocrit; MCH: mean corpuscular hemoglobin; MCV: mean corpuscular volume; MCHC: mean corpuscular hemoglobin concentration; RDW: red cell distribution width

Variables	Cases, mean ± SD	Controls, mean ± SD	Test applied (statistic)	P-value
Hb, gm/dl	10.8 ± 1.34	11.2 ± 1.33	Independent t-test* (-2.07)	0.039
RBC, 10^6^/cc	4.41 ± 0.55	4.42 ± 0.53	Mann-Whitney U (3150)	0.762
HCT, %	33.7 ± 3.72	34.6 ± 3.73	Mann-Whitney U (2867)	0.208
MCH, pg	24.6 ± 2.58	25.4 ± 3.08	Mann-Whitney U (2498)	0.012
MCV, fl	75.5 ± 7.98	78.7 ± 8.06	Mann-Whitney U (2517)	0.015
MCHC, %	31.8 ± 1.68	32.3 ± 1.26	Mann-Whitney U (2455)	0.007
RDW, %	16.6 ± 2.75	14.9 ± 2.0	Mann-Whitney U (1215)	<0.001

## Discussion

Our findings provide valuable insights into the association between IDA and FC among children aged 6-60 months admitted to a tertiary care hospital in West Bengal. The observed higher prevalence of IDA in children with FC compared to febrile children without convulsions supports the hypothesis that iron status may play a key role in the occurrence of FC. The association between IDA and FC aligns with previous research in pediatric populations. A meta-analysis conducted by Kwak et al. in 2017 suggested that IDA is associated with an increased risk of FC among children [5]. Similarly, a study published by Chaudhury et al. [9] in 2021 also demonstrated a similar correlation, emphasizing the importance of proactive screening and management of IDA to potentially reduce the incidence of FC.

The mean hemoglobin levels in the case group were significantly lower than those in the control group, indicating a direct relationship between lower hemoglobin concentrations and the occurrence of FC. This finding echoes the results of a study by Sherjil et al. [11] in 2010. Most studies in the current literature have used serum iron, TIBC, and ferritin levels to confirm the status of IDA. A study conducted by Daoud et al. [12] evaluated the significance of iron status as a possible risk factor for FC. They found that the mean serum ferritin level among cases was 29.5 mcg/L, significantly lower than that among controls (53.5 mcg/L). Similar results were observed in a study from Mumbai by Vaswani et al. [13], where the mean serum ferritin level was significantly lower among patients with first-episode febrile seizures (31.9 ± 31.0 mcg/L) compared to controls (53.9 ± 56.5 mcg/L) (p=0.003).

However, these iron kinetics studies, such as serum iron, ferritin, and TIBC, are often not readily available in many semi-urban and rural areas of most developing countries, and they can be costly procedures. As an alternative, low hemoglobin levels and the triad of low MCH, MCV, and MCHC [10] can be considered surrogate markers for these iron kinetics studies in diagnosing IDA. We incorporated these parameters into our study. Furthermore, in the analysis of RBC indices, our study revealed that MCH, MCV, and MCHC were all significantly lower among cases than in the control group, while RDW was significantly higher among cases than in the control group. These findings are consistent with a study by Ghasemi et al. [14] in 2014, which emphasized the significance of microcytosis and hypochromia in the etiology of FC.

The main strengths of our study lie in the fact that we used standardized criteria for diagnosing IDA and FC, ensured concurrent enrollment of cases and controls, and made sure that there was no recall bias regarding the exposure (IDA) to be studied. While the current study contributes to the growing body of literature on the association between IDA and FC, it is essential to acknowledge its limitations. The cross-sectional nature of the study and its single-center design may limit the generalizability of its findings. Additionally, other potential confounding factors, such as genetic predisposition and infectious etiologies, were not comprehensively explored, warranting further investigations in future research.

## Conclusions

Our findings suggest a significant association between IDA and FC in children aged 6-60 months. These results underscore the importance of considering iron status in clinical management and preventive strategies for FC in pediatric populations. Further multi-center prospective studies are warranted to validate these findings and explore the underlying mechanisms of this association.
